# Alcohol Devitalization and Replantation for Primary Malignant Bone Tumors of the Knee Joint

**Published:** 2017-10

**Authors:** Xihai ZHANG, Ge CHEN, Jun WANG, Lian TANG, Yiran YIN

**Affiliations:** Dept. of Orthopedics, Affiliated Hospital of Southwest Medical University, Luzhou, PR China

**Keywords:** Alcohol-devitalized tumor, Bone replantation, Prosthesis, Bone tumor, Limb function score

## Abstract

**Background:**

This paper is aimed at studying the therapeutic effects of *in situ* replantation of alcohol-devitalized bone segments to treat malignant bone tumors of the knee joint.

**Methods:**

We retrospectively analyzed clinical data for 45 patients from January 2013 to January 2016 who underwent replantation following alcohol-devitalization of bone segments and 40 who underwent prosthesis implantation. The two groups were comparable in basal clinical biometric data, including gender, age, tumor type and location, Enneking staging, and maximum tumor diameter. Radical tumor resection was combined with neoadjuvant chemotherapy following the two-implantation procedures.

**Results:**

The median follow-up time was 25 months, and the outcomes were compared. We found no differences in the length of bone lesions, surgery time, intraoperative blood loss, amount of postoperative drainage, and perioperative complications, which were just three for each method. We also found no significant differences in limb function scores, internal fixation imaging scores, tumor-free survival rate, and overall survival rate between the two groups. Replantation following alcohol-devitalization of tumor-bearing bone segment demonstrated similar clinical outcomes compared with prosthesis implantation in the treatment of primary malignant bone tumors of the knee joint.

**Conclusion:**

Both therapies enjoy good application safety and effectiveness. Because alcohol devitalization is inexpensive and easy to apply in the clinic, it should be considered a preferred method in the treatment of bone tumors.

## Introduction

Thirty to 50% of all bone tumors are primary malignant bone tumors around the knee joint (1). Early surgical resection combined with other comprehensive treatment methods such as neoadjuvant radiotherapy and chemotherapy can salvage the limb in 90 – 95% of the patients. This approach has recurrence and tumor-free survival rates comparable to amputation surgery, but the life quality of patients is significantly improved (2).

There are three kinds of reconstruction methods for bone defects: artificial prosthesis, allogeneic bone transplant, and autologous devitalized bone transplant. The use of prosthesis has gradually increased, but the main complications include infections, loss of internal fixation, and fracture (3). The allogeneic bone transplant is limited by source, rejection, and low patient acceptance (4). Autologous bone can avoid some of these problems and devitalization can be achieved by radiation, high pressure, Pasteurization, liquid nitrogen, and ethanol (5). Two groups used high-pressure steam and liquid nitrogen freezing to devitalize autologous tumor bones (6, 7), which were deemed clinically valuable. Ethanol devitalization has the advantage of being safe, cost-efficient, and shape matching (8).

Here, we employed alcohol devitalization and replantation for the treatment of primary malignant bone tumors around the knee joint, and compared the outcomes with prosthesis replantation. We expect that this study will serve as reference for the rational selection of clinical therapy for bone tumors.

## Methods

### Patient information

We retrospectively examined 85 patients pathologically diagnosed with primary malignant bone tumors around the knee joint from January 2013 to January 2016 in Affiliated Hospital of Southwest Medical University.

Informed consent was taken from the patients and the hospital confirmed ethically the study.

Exclusion criteria were as follows: severe osteoporosis, pathologic fracture, knee arthritis, autoimmune diseases; severe underlying diseases, intolerable to risks from surgery and anesthesia, poor compliance, or incomplete clinical data. This study was approved by the ethics committee of Affiliated Hospital of Southwest Medical University. Signed written informed consents were obtained from the patients and/or guardians. Patients were divided into two groups: alcoholic devitalization / replantation (n = 45) and prosthesis implantation (n = 40). The two groups were comparable regarding gender, age, type and location of the tumor, Enneking staging, and maximum diameter (Table 1).

**Table 1:** Baseline data between the two groups

***Group***	***# cases***	***Male/Female***	***Age (yr)***	***Osteosarcoma***	***Ewing's sarcoma***	***Rhabdomyosarcoma***	***Chondrosarcoma***	***Proximal tibia segment***	***Distal femur segment***	***Middle femur segment***	***Enneking staging Phase IIb***	***Phase III***	***Maximum diameter (cm)***
Alcoholic devitalization	45	32/13	36.5 ± 13.6	22	10	8	5	18	13	14	36	9	5.4 ± 1.3
Prosthesis	40	25/15	38.2 ± 15.4	20	10	7	3	15	16	9	33	7	5.6 ± 1.5
t/χ^2^		0.711	0.352	0.373				1.381			0.087		0.242
*P*		0.399	0.765	0.946				0.501			0.769		0.863

### Surgical methods

The surgeries were conducted by the same surgery and nursing team. Preoperative MRI examination was used to determine that the tumor did not cross the epiphyseal line, and did not invade the subchondral bone. Neoadjuvant chemotherapy regimens included DIA (DDP + ADM + IFO), MMIA (HD + MTX + ADR + IFO), and CHOP (CTX + ADM + VCR + PDN). Artificial prosthesis replantation was carried out according to the standard surgical procedures.

We describe here the distal femur segment as an example to demonstrate the main procedures of replantation following alcohol devitalization of tumor-bearing segments. Overall, 1,500 to 2,500 ml of 99% alcohol was prepared for further use. Patients were placed in supine position under general anesthesia, with pneumatic tourniquet fixed at the thigh root.

1. Exposure of the tumor localization. A longitudinal arc incision about 30 cm long is made at the anteromedial aspect of the knee joint to cut open layer by layer, exposing successively the rectus femoris, vastus medialis, and vastus intermedius to the distal femur segment. Cut open the structure around the knee joint to expose the distal femur segment clearly. Keep the Insall line away from the articular surface for osteotomy.
2. Resection of the tumor bone at the distal femur segment. The surrounding soft tissues are protected with retractors placed on each side of the femur. The periosteum is dissected 2 to 3 cm from the proximal end of the tumor to undergo subperiosteal dissection. Avoid being far from the dissection. Then, cut off the femur with a wire saw, dissect the posterior periosteum, and separate the posterior femoral arteries, veins, and nerves to extend to the popliteal fossa. Vessels within the tumor were cut off with thread. Pull the distal stump of the femur forward and buckle the knee joint to expose fully the posterior femoral soft tissues. Finally, detach the attachment points of the distal femoral muscle as well as the medial and lateral heads of gastrocnemius. Intraoperative exposure is done with the aid of C-arm X-ray. Determine the distal epiphysis and metaphyseal epiphyseal plate. Distal femoral osteotomy plane is located within 5 mm at the lower edge of the tumor. Electric saw is used to break off the femur. Determine the infiltration of the tumor at the end of osteotomy and confirm the existence of tumor cell residue under microscopic cytology. Slowly release the tourniquet, and stop the bleeding completely. Tumor bed is soaked in distilled water at 42°C for 30 min to eliminate tumor deposit.
3. In vitro devitalization of the tumor bone. Open the bone marrow cavity of the excised segment bearing the tumor. Design the screw channel for fixation before devitalization with 99% alcohol for 30 min.
4. *In situ* replantation of bone. Inject pressurized bone cement to the devitalizated bone and conduct *in situ* replantation. Use intramedullary nails for fixation. The epiphysis is fixed by crossing screws with the retained joint surface. If the area of the bone lesion is large, a crimped K-shaped nail may be used to support. The location of the lesion is filled with adriamycin-containing bone cement. If necessary, an autologous iliac bone graft may be placed at the junction of the devitalized bone and host bone to form an extra-cortical bone bridge. After repeated flushing, close by suturing layer by layer. Place a drainage tube and fix it by a long plaster or lower limb brace. Routine antibiotics are administered to prevent infection and the drainage tube can be removed if drainage is less than 50 ml/d. Determine the functional exercise time of lower extremity. Stitches can be removed 14 days later and at that point chemotherapy can be started. External fixation can be removed 8 weeks later. Start knee joint rehabilitation using crutches for about 4 months.

### Observed indicators

Length of bone lesions, surgery time, intraoperative blood loss, amount of postoperative drainage, and perioperative complications were compared between the two groups. The follow-up time was 6 to 41 months with a median time of 25 months. Limb function scores (MSTS) and internal fixation imaging scores (ISOLS) were also compared. MSTS scores include six items: pain, function, psychological tolerance, support, walking, and gait, with a highest score of 5 points for each indicating better function. ISOLS scores include nine items: bone reconstruction, anchoring, interface, fusion, implant, absorption, shortening, fracture, and internal fixation, with a highest score of 4 points for each indicating better performance. The rates of tumor-free survival and overall survival were also compared.

### Statistical methods

Data with normal distribution were expressed as mean ± standard deviation, and compared by t-test. Enumeration data were expressed as percentages and tested with χ^2^. The difference was deemed as statistically significant if *P* <0.05. SPSS20.0 (Chicago, IL, USA) was used for analysis.

## Results

### Length of bone lesions, surgery time, intra-operative blood loss, and postoperative drainage

We compared a series of relevant clinical and surgical parameters between the two groups. We found no significant differences in the length of bone lesions, surgery time, intraoperative blood loss, and amount of postoperative drainage between the two groups surgical limb implantation (Table 2).

**Table 2:** Length of bone lesions, surgery time, intraoperative blood loss and postoperative drainage

***Group***	***Length of bone lesions (cm)***	***Surgery time (min)***	***Intraoperative blood loss (ml)***	***Postoperative drainage (ml)***
Alcoholic devitalization	9.5 ± 1.4	135.4 ± 23.7	126.3 ± 24.9	246.5 ± 42.3
Prosthesis	10.2 ± 1.6	152.3 ± 30.5	132.4 ± 26.7	224.3 ± 45.1
*t*	0.423	0.365	0.385	0.276
*P*	0.649	0.812	0.767	0.865

### Perioperative complications

We observed only three postoperative complications for each procedure (Table 3). The alcohol devitalization group had one infection, one limb shortening and one fracture. The prosthesis group experienced one infection, one loosening of internal fixation, and one fracture. Overall, there was no significant difference in perioperative complications between the two regimens (Table 3).

**Table 3:** Perioperative complications: case (%)

***Group***	***Cases***	***Infection***	***Limb shortening***	***Loose internal fixation, dislocation, breakage***	***Fracture***	***Incidence of complications***
Alcoholic devitalization	45	1	1	0	1	3 (6.7)
Prosthesis	40	0	1	1	1	3 (7.5)
χ^2^						0.000
P						1.000

### Follow-up MSTS and ISOLS scores

We next compared the functional (MSTS) and imaging (ISOLS) scores during follow up. The functional scores were slightly higher in the prosthesis group whereas the imaging scores were slightly higher in the devitalization group. However, there was no significant statistical difference for MSTS and ISOLS scores between the two groups (Table 4).

**Table 4:** Follow-up MSTS and ISOLS scores

***Group***	***MSTS***	***ISOLS***
Alcoholic devitalization	24.3 ± 5.6	32.2 ± 4.9
Prosthesis	25.2 ± 5.8	31.6 ± 5.1
*t*	0.264	0.213
*P*	0.822	0.865

### Tumor-free and overall survival

Finally, we analyzed the tumor recurrence and overall survival port-surgery. Both tumor-free and overall survival were slightly higher in the devitalization procedure. However, we found no significant differences in the tumor-free survival rate and overall survival rate between the two groups (Table 5).

**Table 5:** Rates of tumor-free survival and overall survival: case (%)

***Group***	***# of cases***	***Tumor-free survival***	***Overall survival***
Alcoholic devitalization	45	38 (84.4)	42 (93.3)
Prosthesis	40	36 (90.0)	38 (95.0)
χ^2^		0.580	0.000
*P*		0.446	1.000

## Discussion

In devitalization and replantation with preservation of the joint, it is important to preserve the articular cartilage surface and at least 1 cm of partial subchondral bone. Based on that, extended resection of the tumor-bearing segment is conducted, followed by devitalization for at least 30 min, *in situ* implantation, and internal fixation to restore the continuity and integrity of the adjacent joints. The long-term outcomes are similar for marginal and wide excision of osteosarcoma (9), which can maximize the preservation of healthy tissues such as ligaments and tendons and help with postoperative rehabilitation. Ethanol can denature and kill the tumor shell.

Devitalized bone can still restore the continuity of bone cortex and backbone, reduce the adverse effects on activity and biomechanical properties of the bone tissues, and promote bone reconstruction (10). In addition, the devitalized tumor cells can act as antigen molecules to stimulate effectively the immune response and to increase cellular immune function (11). Preservation of articular cartilage plays an important role in enhancing the stability of the articular surface, significantly reducing the complications of artificial prosthesis such as wear, fracture, and loosening (12). Alcohol devitalization / replantation can significantly improve the limb salvage rate. The limb can survive for a long time even with a tumor, significantly prolonging the life of the prosthesis or amputation (13). The disadvantages of alcoholi devitalization are prolonged angiogenesis and increased incidence of delayed healing or no healing of the bone (14). In addition, there are complications such as local skin wound infection, necrosis, loosening and breakage of internal fixation, insufficient early bone strength, fracture vulnerability, and long-term articular cartilage degeneration (15). Among these, the incidence of devitalized bone fractures is 20 – 30% and breakage of internal fixation 5%–10% (16). Following alcoholi devitalization, a continuous callus can appear in around 8 weeks and the bone can completely heal in 12 weeks (17). The healing modes of the bone after radiation and alcohol devitalization are similar. Both are completed by creeping substitution with an average of 1 cm every 10 months. For the femur, the time from the new bone formation in the host bone to complete bone healing is about 4 to 6 months and for the tibia is 6 – 8 months. Creeping substitution may be the major osteogenic pathway at bone junctions (18).

We summarized our surgical experience as follows: 1. Alcohol devitalization of the tumor is applicable to type I non-pathological fractures located at the bone metaphysis. 2. Confirm by preoperative MRI examination that the tumor does not invade the epiphyseal line to reach the subchondral bone and that the osteotomy plane is within 1 cm from the lower edge of the tumor. 3. Combination of neoadjuvant chemotherapy and extended surgery can be used to eradicate the tumor, which includes 2 – 3 sessions of neoadjuvant chemotherapy and 8 – 10 sessions of postoperative chemotherapy. 4. A drill is used to create a channel prior to alcohol devitalization to facilitate fixation by the crossing screws. 5 During replantation of the devitalized bone, start from bone metaphysis of the preserved joint to the devitalized bone for maximum restoration of the integrity of the devitalized bone shell. 6. The combination of intramedullary nail and crossing screws is preferred for internal fixation. 7. If necessary, wire fixed iliac autograft or allograft bone plate can be used at the osseointegration site between the proximal host and the devitalized bone, where extra-cortical graft will form to promote early bone healing. 8. Postoperative fixation of lower limb by brace or plaster for 3 – 6 months and crutches for 3 to 6 weeks. Early weight-bearing limb exercises are not encouraged to reduce complications such as breakage and dislocation of the internal fixation.

## Conclusion

The clinical outcome of *in situ* replantation of alcohol-devitalized tumor bearing bone segments demonstrates similar clinical outcomes with prosthesis replantation in the treatment of primary malignant tumors of the knee joint. Alcohol-devitalized bone replantation shows good application safety and effectiveness. Alcohol devitalization is inexpensive and user-friendly, and is a good clinical option. The limitations of the study include a small sample size and short follow-up time, which need further verification.

## Ethical considerations

Ethical issues (Including plagiarism, informed consent, misconduct, data fabrication and/or falsification, double publication and/or submission, redundancy, etc.) have been completely observed by the authors.
